# Editorial: Emerging talents in Frontiers in pharmacology: Gastrointestinal and hepatic pharmacology 2022

**DOI:** 10.3389/fphar.2022.1055030

**Published:** 2022-10-21

**Authors:** Laura Grasa, Thomas Brzozowski, Marilia Seelaender

**Affiliations:** ^1^ Department of Pharmacology, Physiology and Legal and Forensic Medicine, University of Zaragoza, Zaragoza, Spain; ^2^ Department of Physiology, Jagiellonian University Medical College, Krakow, Poland; ^3^ Department of Surgery and LIM 25-HC, Faculdade de Medicina, University of São Paulo, São Paulo, Brazil

**Keywords:** inflammatory bowel disease (IBD), irritable bowel syndrome (IBS), liver disease, cancer, pancreatitis

This special edition of *Frontiers in Pharmacology* is dedicated to students from all over the world that undertake key research as part of education in Gastrointestinal and Hepatic Pharmacology. Here we present an article collection dedicated to highlighting the emerging talent of student researchers within the field of Gastrointestinal and Hepatic Pharmacology submitted on that occasion.

First, we present some articles related to the pharmacology of gastrointestinal diseases.

Zhang et al. overviewed and extended the discussion on the understanding of ulcerative colitis (UC) and postulated causes and mechanisms, while also providing biometric data on the last decade efforts to examine this disorder. The review stresses the need for collaborative work in Asia. Authors provide an insight into the mechanism of microbiome-induced inflammation, with major focus to NLRP3 inflammasome pathway, which has been one of the most explored and discussed “hot topics” in the scenario of this disease. They emphasized the potential of faecal microbiota transplants, anti-integrin treatment and the use of JAK inhibitor as the nowadays treatment options in patients with UC.

Li et al. performed and described a very diligent study showing that nintedanib, a new drug from the group of tyrosine kinase inhibitors, can be useful in the treatment of inflammatory bowel disease (IBD), by restoring intestinal permeability and the intestinal microbiota. Using advanced bioinformatics analysis and cellular and animal models of intestinal inflammation, they show that the action mechanism of this drug would involve the inhibition of the PCK1 and EFNA1 genes, which are regulated by the transcription factor CEBPB through two super-enhancers (sc-CHR20-57528535 and sc-CHR1-155093980). In addition, nintedanib improves the levels of beneficial microbiota for intestinal health.

Wang et al. presented an important dataset on the UK Biobank collection of over 100,000 adult irritable bowel syndrome (IBS) patients based on the incidence criteria and influencing factors, including pathogenesis and diagnosis criteria. They found that the majority of patients suffered from mixed IBS as the dominant subtype, followed by diarrhea-dominant IBS and constipation-dominant IBS. Precise analysis of the data showed that somatization and celiac disease were the main risk factors for IBS, and that risk factors such as gender differences in mental health are critical to physician diagnosis and treatment of particular IBS subtypes.

Guo et al. have determined the mechanism of cisplatin chemotherapy, which is known to induce nausea and vomiting in cancer patients suffering from depressive mood disorder. They developed experimental two animal models of chronic, unpredictable, mild stress that induced a depression-like phenotype in rats and a model resembling cisplatin-induced vomit, and investigated kaolin and food intake after administration of cisplatin. They found that chronic stress increased 5-HT and SP levels, accompanied by upregulation of 5-HT_3_R, DA_2_R, NK_1_R and downregulation of CB_1_R expression in the core extension. In contrast, chronic stress decreased the expression of 5-HT_3_R, DA_2_R, NK_1_R and increased expression of CB_1_R in the ileum. They concluded that anorexia and vomiting are exacerbated by chronic stress due to the activity of vomit-related molecules and their overexpression in the enteric nervous system (ENS).

Second, we present some articles of talented young researchers related to the pharmacology of liver and gallbladder diseases.

Ma et al. present novel and exciting results on the pharmacological regulation with Empagliflozin (EMPA) of Sestrin 2, a protein induced by stressing stimuli common to obesity-related diseases, such as nonalcoholic fatty liver disease (NAFLD). By performing *in vivo* and *in vitro* experiments, they provide further evidence showing this drug to prevent steatosis and hepatic inflammation. The authors elegantly demonstrate that, by inducing upregulation of Sestrin 2, EMPA modulates cell signaling via the AMPK/mTOR pathway. In addition to unveiling the mechanism of action of EMPA, they propose Sestrin 2 as target for further study of therapeutic strategies aiming at mitigating NAFLD.

Hou et al. presented the data of a clinical trial with regorafenib in the treatment for patients with hepatocellular carcinoma (HCC) who have progressive disease despite anti-tumor treatment with sorafenib. The results of this retrospective analysis in Chinese patients indicate that the second-line oral Regorafenib treatment is safe and can significantly improve overall survival of HCC patients.

Yang et al. have validated a novel model using aminotransferase (ALT) and total bilirubin dynamic evolution patterns to predict acute liver failure (ALF) in patients suffering from drug-induced liver injury. In comparison with other predictive models like Hy´s law or Robles-Diaz Model, this model has significantly higher capability of ALF prediction. In addition, the predictive potency of the model for ALF can be improved incorporating other parameters like the international normalized ratio (INR) and alkaline phosphatase (ALP).

Yan et al. have demonstrated a novel mode of action of the farnesoid X receptor (FXR) during the biliary obstruction. It is known that FXR is a key factor regulating hepatic bile acid synthesis and enterohepatic circulation. However, the authors show, for the first time, that the restoration of the FXR pathway improves both, the intestinal barrier damage and intestinal microbiota imbalance in rats with experimental bile-duct ligation. These findings provide a new, possibly translational potential target for clinical prevention of intestinal mucosal barrier injury in patients with obstructive jaundice.

Finally, we present an experimental study about the treatment of acute pancreatitis.

Yang et al. undertook *in vivo* and *in vitro* studies on the mechanism of the Chinese formula, Chaiqin chengqi decoction (CQCQD) against the development of acute pancreatitis (AP), introducing a new murine model of obesity-induced alcohol-AP. They assessed AP severity and its correlation with pancreas and fat using transcriptomic analysis and network pharmacology, as well as the interactions between CQCQD compounds and their key targets. They found that AP and systemic inflammation were attenuated by CQCQD through the activation of Nrf2/HO-1 antioxidant proteins and significant reduction of PI3K/Akt phosphorylation in pancreatic and adipose murine tissue. Moreover, CQCQD was effective in protecting freshly isolated acinar cells *in vitro* from oxidative stress-induced damage and necrotic cell death. These investigators conclude that CQCQD could alleviate AP severity by activating antioxidant proteins and reducing the PI3K/Akt signaling pathway in the pancreas and visceral adipose tissue associated with obesity.

